# Acute Interstitial Nephritis in a Patient with Non-Small Cell Lung Cancer under Immunotherapy with Nivolumab

**DOI:** 10.1155/2019/3614980

**Published:** 2019-01-15

**Authors:** Panagiotis I. Georgianos, Vasilios Vaios, Eleni Leontaridou, Georgia Karayannopoulou, Triantafyllia Koletsa, Athanasios Sioulis, Elias V. Balaskas, Pantelis E. Zebekakis

**Affiliations:** ^1^Section of Nephrology and Hypertension, 1st Department of Medicine, AHEPA Hospital, Aristotle University of Thessaloniki, Thessaloniki, Greece; ^2^Department of Pathology, Aristotle University of Thessaloniki, Thessaloniki, Greece

## Abstract

Immune-checkpoint-inhibitors (ICPIs) represent a novel class of immunotherapy against several malignancies. These agents are associated with several “immune-mediated” adverse effects, but the reported renal toxicity of ICPIs is less well defined. We present the case of a 60-year-old man with a history of non-small cell lung cancer, who developed acute kidney injury (AKI) approximately 3.5 months after initiation of immunotherapy with nivolumab. Urinalysis revealed sterile pyuria, without microscopic hematuria or proteinuria. Immunological examination was negative. A renal biopsy showed severe interstitial inflammatory infiltration of T-cells, monocytes, and eosinophils without interstitial granulomas and normal appearance of glomeruli, indicating acute interstitial nephritis (AIN) as the cause of AKI. After a short-term course of corticosteroids and permanent nivolumab discontinuation, partial recovery of renal function was noted. AIN is a rare adverse effect of ICPIs that mandates the close monitoring of renal function in patients under immunotherapy with these agents.

## 1. Introduction

Immune-checkpoint-inhibitors (ICPIs) are a newly introduced class of immunotherapy against several solid organ and haematological malignancies [1]. These agents are monoclonal antibodies targeting anticytotoxic T-lymphocyte-associated protein 4 (CTLA-4) and antiprogrammed death 1 (PD-1) signalling pathways, which act as negative immunologic regulators on T-cells and other immune cells [1]. Inhibition of these pathways activates tumor-directed immune responses, engaging the patient's own innate and adaptive immune system against tumour cells [1].

ICPIs have been associated with a number of unique “immune-related” adverse reactions, possibly mediated through nonspecific immune activation against self-antigens [2]. The most commonly reported ICPI-related adverse events include skin rash, colitis, hepatitis, hypophysitis, and other endocrinopathies [2]. Although these agents were originally considered as nonnephrotoxic, a growing body of evidence suggests that ICPIs are associated with increased risk for acute kidney injury (AKI), glomerular damage, and electrolyte disturbances [3–6]. However, the clinical spectrum, pathogenesis, and therapeutic approach of ICPI-associated AKI still remain elusive.

Herein, we present the case of a biopsy-proven acute interstitial nephritis (AIN) in a 60-year-old man with a history of non-small cell lung cancer under immunotherapy with the PD-1 inhibitor nivolumab.

## 2. Case Presentation

A 60-year-old man was admitted to our department due to progressive deterioration of renal function approximately 3.5 months after initiation of immunotherapy with nivolumab. In April 2016, there was a diagnosis of stage IIIa non-small cell lung cancer located in the upper lobe of right lung was made (cT3N2M0). Lung cancer was initially treated with combination of radiotherapy and 6 cycles of chemotherapy, including paclitaxel and carboplatin. In March 2017, a positron-emission-tomography/computed-tomography (PET/CT) scan showed malignant extension to tracheobronchial and subcarinal lymph nodes. Immunotherapy with nivolumab was initiated at a dosing regimen of 3 mg/kg every 2 weeks. Immunotherapy started with a normal renal function (serum creatinine: 79.56 *μ*mol/l, estimated-glomerular-filtration-rate (eGFR): 92.5 ml/min/1.73m^2^). After the 7th infusion of nivolumab (approximately 105 days after initial exposure), laboratory examinations revealed for first time impaired renal function (serum creatinine: 176.8 *μ*mol/L, eGFR: 35.2 ml/min/1.73m^2^). Treating oncologists decided the administration of 2 additional cycles of nivolumab with progressive doubling of serum creatinine and eGFR decline to 14.8 ml/min/1.73m^2^ before referral to the renal department (Table 1).

On admission, the patient's medical history revealed that he was a former heavy smoker over the past 35 years (20 cigarettes per day) and had no other comorbidities. He did not receive any medications with the exception of sporadic use of simple analgesics. He denied the use of nonsteroidal anti-inflammatory drugs, proton pump inhibitors, or other nephrotoxic agents, and he reported no drug or food allergies. His family history was unremarkable. The physical examination revealed a normal body temperature (36.7°C), blood pressure 135/70 mmHg, pulse rate 80 bpm, oxygen saturation 98% in the room air, and absence of abnormal clinical signs from the chest auscultation and palpation of the abdomen. Pedal edema, skin rash, joint pain, and swelling were not present. Blood tests revealed mild anemia (hemoglobin: 12.0 g/dl), severely impaired renal function (serum creatinine: 433.1 *μ*mol/L, eGFR: 11.9 ml/min/1.73m^2^), and hyperkalemia (serum potassium: 5.8 mmol/L) with no other electrolyte or acid-base disturbances. Urinalysis showed sterile pyuria and absence of both proteinuria and microscopic hematuria. A 24-hour urine collection confirmed the absence of proteinuria. Renal ultrasonography excluded the presence of hydronephrosis and showed kidneys with normal size, contour, and cortical echotexture.

**Figure 1:**
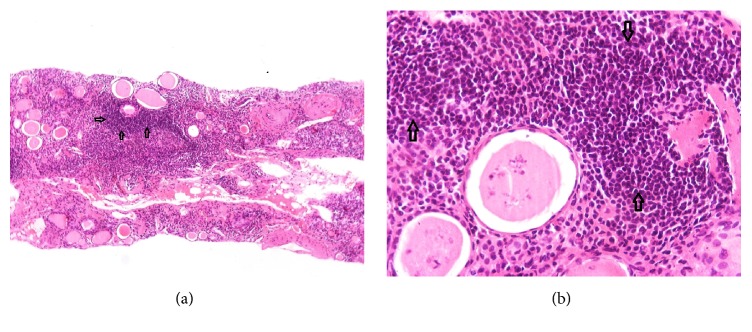
(a) Hematoxylin and eosin stain show typical histopathological appearance of acute interstitial lesions in light microscopy. (b) Hematoxylin and eosin stain shows diffusive interstitial infiltration of polymorphic inflammatory cells, predominantly T-cells, monocytes, and eosinophils.

With respect to the diagnostic work-up of AKI, screening for hepatitis B and C viruses and HIV was negative. Immunological tests including antinuclear and anti-DNA antibodies, rheumatoid factor, anti-neutrophil cytoplasmic autoantibodies (ANCA), complement and serum immunoglobin levels were negative or within the normal range. Electrophoresis and immunofixation did not identify the presence of a monoclonal immunoglobin component in the serum. The absence of both proteinuria and microscopic hematuria and the negative immunological examination raised the clinical suspicion of AIN and a renal biopsy was performed to ascertain the cause of AKI. Light microscopy showed severe interstitial nephritis with infiltration of polymorphic inflammatory cells (Figure 1(a)). The interstitial inflammatory infiltrate was predominantly composed of T cells, monocytes, and eosinophils. Inflammatory infiltrates were also present in the tubular basement, and tubular epithelial cells exhibited diffusive degenerative lesions; interstitial granulomas were not present (Figure 1(b)). Glomeruli were normal, except for 2 out of 12, which were fully sclerotic. Immunofluorescence was negative for glomerular or tubular immune deposits.

After the biopsy-proven diagnosis of AIN, the patient received 500 mg/day intravenous methylprednisolone for 3 days, followed by oral prednisolone 1.0 mg/kg/day for the subsequent 2 weeks. Prednisolone dose was progressively tapered and the total duration of corticosteroid therapy was 8 weeks. Immunotherapy with nivolumab was permanently withdrawn. The patient was followed up closely in the Outpatient Nephrology Clinic (Table 1). At month 2 after discharge, renal function was significantly improved (serum creatinine: 194.5 *μ*mol/L, eGFR: 31.4 ml/min/1.73m^2^) and then stabilized at 6-month follow-up visit to a slightly higher eGFR level, suggesting a partial, but clinically meaningful, recovery of kidney injury.

## 3. Discussion

This case report highlights that AIN is a serious ICPI-induced renal adverse event that mandates the close monitoring of renal function in patients receiving immunotherapy with these agents. Any deterioration in renal function should always raise the suspicion of ICPI-associated nephrotoxicity, particularly in the absence of background therapy with other agents that may induce AIN, as in the patient of our case report. Early recognition of this rare nephrotoxic reaction by treating oncologists may be of major importance for the subsequent clinical course and recovery from AKI.

In a review of renal toxicities reported to the Food and Drug Administration adverse reporting database (FAERS) between the 3rd quarter of 2011 and 1st quarter of 2015, Wanchoo et al. [2] identified 20 cases of renal impairment and another 24 cases of serious electrolyte disturbances attributable to nivolumab use. By analyzing clinical-trial data from 3,695 patients, Cortazar et al. [7] reported an overall incidence of ICPI-associated AKI as high as 2.2%. In a 2018 meta-analysis of 48 randomized trials (incorporating data from 11,845 participants), compared with controls receiving nonnephrotoxic agents, patients treated with PD-1 inhibitors had a 4.2-fold higher risk of AKI [Relative Risk (RR): 4.19; 95% Confidence Interval (CI): 1.57-11.18] [5]. The pooled incidence of AKI among patients treated with PD-1 inhibitors was 2.2% [5]. It has to be noted, however, that these estimates may underestimate or overestimate the true burden of ICPI-inducible nephrotoxicity, given the limitations related to adverse event reporting in clinical trials, the heterogeneity in definitions of AKI across studies, and rarity of biopsy-proven cases that may provide more causal associations. A summary of previously reported cases of nivolumab-associated nephrotoxicity is provided in Table 2 [4, 8–13].

In the patient of our case, the time elapsed from the initial exposure to nivolumab until the onset of AKI was 105 days. This delayed response is in line with the observations of a case-series of 13 patients with ICPI-induced AKI, in which the median time from initiation of an ICPI to AKI was 91 days (range: 21-245 days) [7]. The heterogeneous and delayed onset of AKI is suggestive of a mechanistic pathway distinct from the typical drug-induced hypersensitivity reaction. ICPI-associated AIN is proposed to be mediated through “reprogramming” of the innate and adaptive immune system, a process resulting in loss of immune tolerance against self-antigens at the kidney level [14, 15]. This hypothesis is supported by experimental studies showing that the PD-1 signalling pathway is expressed in tubular epithelial cells and exerts protective actions against T-cell-mediated autoimmunity [14, 15]. Animal studies showed that PD-1 knockout mice were prone to developing AIN and glomerulonephritis [16]. Similarly, administration of anti-PD-1 monoclonal antibody in mice was able to induce interstitial and glomerular damage, possibly mediated through autoimmune overactivity [17]. An alternative mechanistic explanation is that ICPIs may induce suppression of T-cell immunity that results in loss of normal renal tolerance against concomitantly administered drugs known to be associated with AIN [11, 13, 18]. This is a less likely explanation for pathogenesis of AIN in our case presentation, since the patient did not have comorbidities and denied the use of nonsteroidal anti-inflammatory drugs, proton pump inhibitors, or other nephrotoxic agents.

The efficacy of corticosteroids in remission of kidney injury is another area surrounded by controversy in the therapeutic approach of AIN management. A short-term course of corticosteroids is recommended as an approach to hasten the recovery of renal function in biopsy-proven AIN, particularly in patients requiring support with hemodialysis or when withdrawal of the inciting agent is not accompanied by the anticipated improvement in renal function [19, 20]. In the aforementioned case-series of Cortazar et al. [7] complete or partial remission of renal damage was observed in 9 out of 10 patients with AIN who received short-term corticosteroid therapy; in contrast, 2 patients with AIN not given corticosteroids had no improvement in renal function [7]. Undoubtedly, the observational nature of these findings cannot provide cause-and-effect associations, and the efficacy and safety of corticosteroids warrant evaluation in properly designed randomized trials. In addition, duration of AIN and severity of interstitial fibrosis in kidney biopsy are shown to be strong predictors of natural course of AIN [21]. The long duration of AIN, diffusive interstitial inflammatory infiltrate, and severity of tubular lesions that were evident in the kidney biopsy may be plausible explanations for the only partial recovery of renal function in response to corticosteroid therapy in our case presentation.

Although immune checkpoint inhibitors were originally considered as nonnephrotoxic immunotherapy against solid organ and haematological malignancies, this case-report highlights that acute interstitial nephritis is a rare, but serious, adverse effect of nivolumab. A short course of corticosteroid therapy is a promising therapeutic approach in order to achieve complete or partial remission of nivolumab-induced AIN. Close monitoring of renal function and serum electrolyte balance is required for early recognition of renal toxicities associated with ICPI use in daily clinical practice.

## Figures and Tables

**Table 1:** Patient laboratory value after initiation of immunotherapy with nivolumab.

**Parameter**	**Nivolumab therapy**			**Nivolumab withdrawal**				
	**Day 0**	**Day 105**	**Day 130**	**Admission**	**Discharge**	**Month 1**	**Month 2**	**Month 6**
Serum creatinine (*μ*mol/L)	79.56	176.8	362.4	433.1	433.2	274.1	194.5	159.1

eGFR^*∗*^ (ml/min/1.73m^2^)	92.5	35,2	14,8	11.9	14.8	20.7	31.4	40

Serum sodium (mmol/L)	-	145	-	138	143	137	140	143

Serum potassium (mmol/L)	-	5.3	-	5.8	4.3	5.4	4.5	4.7

Serum calcium (mmol/L)	-	2.45	-	2.2	2.2	2.4	2.2	2.25

Serum phosphate (mmol/L)	-	-	-	1.35	1.32	1.42	0.97	1.23

Eosinophil count (cells/*μ*L, %)	-	-		320 (6.9%)	260 (4.0%)	230 (3.7%)	80 (0.9%)	50, (0.5%)

CRP (nmol/L)	-	-	-	780.0	207.6	138.1	85.7	57.1

UPE (mg/day)	-	-	-	180	-	190	201	266

CRP= C-reactive protein; eGFR= estimated glomerular filtration rate; UPE= urine protein excretion.

^*∗*^CKD-EPI equation was used to estimate eGFR

**Table 2:** Case reports associating nivolumab therapy with nephrotoxic reactions.

**Author**	**Patients**	**Age**	**Gender**	**ICPI**	**Cancer**	**Renal biopsy**	**Treatment**	**Outcome**
Hoffman et al. [9]	1	52 yrs	M	Nivolumab	Melanoma	Not available	Steroids	Recovery

Shirali et al. [13]	6	67.5 yrs	3M/3F	Nivolumab/ Pembrolizumab/ Ipilimumab	NSCLC	Tubulointerstitial inflammation with infiltrate of lymphocytes	Steroids in 5 of 6 patients	Recovery

Kishi et al. [10]	1	72 yrs	M	Nivolumab	NSCLC	IgA nephropathy	No steroids	Recovery

Murakami et al. [12]	1	75 yrs	M	Combination of Nivolumab and Ipilimumab	Melanoma	Tubulointerstitial inflammation with infiltrate of lymphocytes	Steroids	Recovery

Koda et al. [11]	1	67 yrs	M	Nivolumab	NSCLC	Acute tubulointerstitial nephritis without granuloma formation	Steroids	Recovery

Bottlaender et al. [8]	1	76 yrs	F	Ipilumumab-Nivolumab	Melanoma	Interstitial edema with dense inflammatory infiltrates	Steroids	Recovery

Jung et al. [4]	1	70 yrs	M	Nivolumab	Kidney cancer	Diffusive tubular injury and complex-mediated glomerulonephritis	Steroids and hemodialysis	Recovery

ICPI= immune checkpoint inhibitor; NSCLC= non-small cell lung cancer; M= male, F= female.

## Abbreviations

AIN: Acute interstitial nephritis
AKI: Acute kidney injury
ANCA: Anti-neutrophil cytoplasmic autoantibodies
CI: Confidence interval
CTLA-4: Anticytotoxic T-lymphocyte-associated protein 4
eGFR: Estimated glomerular filtration rate
ICPIs: Immune checkpoint inhibitors
PD-1: Programmed death-1
PET/CT: Positron-emission-tomography/computed-tomography
RR: Relative risk.
